# Evaluation of Macular and Retinal Ganglion Cell Count Estimates for Detecting and Staging Glaucoma

**DOI:** 10.3389/fmed.2021.740761

**Published:** 2021-10-01

**Authors:** Yali Wu, Qing Cun, Yijin Tao, Wenyan Yang, Jia Wei, Daoqing Fan, Ying Zhang, Qin Chen, Hua Zhong

**Affiliations:** ^1^Department of Ophthalmology, The First Affiliated Hospital of USTC, Division of Life Sciences and Medicine, University of Science and Technology of China, Hefei, China; ^2^The First Affiliated Hospital of Kunming Medical University, Kunming, China; ^3^The First Affiliated Hospital of Nanjing Medical University, Nanjing, China

**Keywords:** glaucoma, estimated retinal ganglion cell, standard automated perimetry, retinal nerve fiber layer, ganglion cell complex

## Abstract

**Purpose:** To investigate the clinical significance of macular estimated retinal ganglion cell (mRGC) and estimated retinal ganglion cell (eRGC) in the diagnosis and staging of glaucoma.

**Methods:** This is a cross-section study. All enrolled subjects underwent standard automated perimetry (SAP) and optical coherence tomography (OCT) examination. Swedish Interactive Threshold Algorithm (SITA)-FAST detection strategy and 24-2, 10-2 detection programs were employed in SAP assessment. The visual-field parameters and OCT parameters were calculated according to three formulas to obtain the eRGC and mRGC1 or mRGC2. The efficiency of eRGC, mRGC1, and mRGC2 estimates for the staging of glaucoma was compared. The sensitivity and specificity of each parameter for diagnosis of glaucoma were analyzed using the receiver operating characteristic (ROC) curve.

**Results:** A total of 119 eyes were included in the analysis. Compared with the healthy controls, eRGC, mRGC1, and mRGC2 estimates were significantly decreased in patients with glaucoma. As glaucoma progressed, eRGC, mRGC1, and mRGC2 estimates were gradually reduced. In preperimetric glaucoma, mRGC1, mRGC2, and eRGC were reduced by 13.2, 14.5, and 18%, respectively. In the mild stage of glaucoma, mRGC1, mRGC2, and eRGC were reduced by 28, 34, and 38%, respectively. In the advanced stage of glaucoma, mRGC1, mRGC2, and eRGC were reduced by 81, 85, and 92% respectively. The proportion of retinal ganglion cell (RGC) loss in the macula was close to that outside the macula. The specificity at 95% gave a sensitivity of 95.51, 86.52, and 87.64% for eRGC, mRGC1, and mRGC2, respectively. The sensitivity of structural parameters macular ganglion cell complex thickness and retinal nerve fiber layer (RNFL) were 98.88 and 95.51%, respectively. The sensitivity of functional parameters mean deviation (24-2) and visual field index (VFI) were 80.90 and 73.03%, respectively. The area under ROC curve of mRGC1, mRGC2, and eRGC were 0.982, 0.972, and 0.995 (*P* < 0.0001), respectively.

**Conclusion:** Estimated retinal ganglion cell, mRGC1, and mRGC2 provide value to the staging of glaucoma and better diagnostic performance. Macular RGC estimatesthat integration of both structural and functional damages in macular may serve as a sensitive indicator for assessing macular damage in glaucoma and are of importance for the diagnosis and progression management of glaucoma.

## Introduction

A glaucoma is a group of progressive optic neuropathies characterized by thinning of the retinal nerve fiber layer (RNFL) and cupping of the optic disc (1). Such structural changes are usually accompanied by functional impairments, which may eventually lead to irreversible vision loss. Both the characteristic structural and functional changes are related to pathological loss of retinal ganglion cell (RGC) somas and axons (2). Clinical measurements of structural and functional damage in glaucoma are currently using standard automated perimetry (SAP) and optical coherence tomography (OCT), respectively, for diagnosis and staging of disease. However, these measurements of structural and functional changes are variable and inconsistently related to one another in many cases. For instance, studies have shown that visual field changes precede neural structural loss in mild stages of diseases (3–11); on the contrary, evidence is found that visual field changes are only detected when damages to the nerve fiber layer reach 40% or more (6, 7, 12, 13). Such common inconsistency between structural and functional measurements is largely considered attributed to different measurement scales and computational methods. Therefore, single structural or functional measures are not enough to identify and monitor progressive glaucomatous damage.

Harwerth et al. demonstrated that structural and functional tests are in agreement as long as one uses appropriate measurement scales for neural and sensitivity losses and takes the effect of aging and eccentricity into account on estimates of neural losses. Consecutive studies have found that the estimates of RGC losses from visual field detection using perimetry closely fit that from RNFL detection using OCT (14). In addition, ganglion cell complex (GCC) thickness had been recognized as an important indicator for glaucoma. Based on the original RGC estimation model proposed by Harwerth et al. (14, 15) and Medeiros et al. (16, 17), we incorporated the macular RGC estimation model (18) and made appropriate adaptations. The structural evaluation was modified and peripapillary RNFL thickness measurements were replaced by macular structural parameter GCC. SAP program 24-2 (6° interval) was changed to program 10-2 (2° interval). We sought to integrate macular structural and functional parameters to obtain macular estimated retinal ganglion cell (mRGC) and to determine whether these parameters are superior to structural or functional analysis individually in the discrimination of glaucomatous from healthy eyes and its performance in staging the disease.

## Method

### Study Design and Participants

This was an observational, cross-sectional study. The study has been approved by the Ethics Committee of the First Affiliated Hospital of Kunming Medical University and was implemented following the Declaration of Helsinki. All participants gave written informed consent before the study. All patients underwent a detailed and comprehensive medical history and ophthalmologic examinations, namely, ophthalmic routine slit-lamp examination, diopter examination, anterior chamber angle, and fundus photography, A-scan measures central corneal thickness and axial length, Goldmann intraocular pressure (IOP), and average RNFL thickness, and visual field examination. A total of 89 eyes of patients diagnosed with primary open-angle glaucoma (POAG) in the First Affiliated Hospital of Kunming Medical University from February 2015 to February 2017 were included, according to the Advanced Glaucoma Intervention Study (AGIS) visual field scoring system, 20 eyes with preperimetric glaucoma, 20 eyes with the mild stage of disease (1–5 points), 20 eyes with the moderate stage (6–12 points), and 29 eyes with the severe stage (13–20 points). The normal control group includes 30 eyes of healthy examined subjects at the same period. Inclusion criteria were: the best-corrected visual acuity was not less than 20/40, spherical refraction ranged from −5 to +5 D, cylinder correction ranged from −3 to +3 D, and subjects were excluded if they presented with nonglaucomatous visual field defective diseases, such as optic neuritis, history of ocular trauma, macular lesions, or intraocular surgery.

### Visual Field Testing

All subjects were examined with the Humphrey Visual Field Analyzer II (Carl Zeiss Meditec, Dublin, CA, USA) using Swedish Interactive Threshold Algorithm (SITA)-FAST 24-2 and 10-2 programs. Each visual field test was performed by the same physician. Visual fields with a solid fixation loss rate of <20%, a false-negative rate of <33%, and a false-positive rate of <15% were considered reliable results. The visual field that the false-negative rate was higher than 33% but indicative of severe disease progression (mean deviation (MD) value below −12 dB) was also considered for the calculation. Repeated visual field examinations were required if eyelid masking, fatigue, incorrect fixation, or learning effects were present.

### Optical Coherence Tomography

Subjects underwent optic nerve head (ONH) scanning program for ONH examination and GCC program for macula using the ultrahigh speed 70 kHz Fourier domain OCT system (RTVue XR Avanti, Optovue, Inc., Fremont, CA, USA). Peripapillary RNFL and GCC thickness probability maps with eight partitions were obtained by measuring the RNFL thickness of the optic disc and the macular GCC thickness. High-quality images with signal intensity index >50 without motion and vitreous floaters were considered for analysis. OCT testing and visual field testing were performed on the same day.

### Integrated Structure-Function Estimate of RGC Counts

Harwerth et al. (19) proposed an empirical formula for estimating the counts of corresponding RGCs based on experimental studies on rhesus monkeys, namely, SAPrgcs from the visual field (Humphrey 24-2) detection as function measurements and OCTrgc obtained OCT detection as peripapillary structure measurements. The experimental model was translated to clinical perimetry in humans and has been validated in glaucoma and normal IOP glaucoma (5, 14, 20). Considering the effects of aging on axonal degeneration and varying severity of the disease, factors such as age and MD have been adjusted in OCTrgc. The use of this model would translate region-specific visual field sensitivity into estimate RGC for the corresponding regions.


$$\begin{array}{l} m=\left[0.054\left(ec\times 1.32\right)\right]+0.9\\ b\;=\left[\;-1.5\left(ec\times 1.32\right)\right]-14.8\\ gc\;=\left\{\left[\left(s-1\right)-b\right]/m\right\}\;+\;4.7\\ SAPrgc=\sum 10ˆ\left(gc\times 0.1\right)\\ d=\left(-0.007\times age\right)+1.4\\ c\;=\;\left(-0.26\times MD\right)+0.12\\ a=\text{average RNFL thickness}\times 10,870\times d\\ OCTrgc=10ˆ\left\{\;\left[\log\left(a\right)\times 10-c\right]\;\times 0.1\right\} \end{array}$$

In the above formulas, *ec* is the retinal eccentricity, which refers to the distance from the central fixation to a certain point on the retina. Based on the results, the values of *ec* were taken separately as 4.2 (3 × 3 deg), 12.8 (9 × 9 deg), 21.2 (15 × 15 deg), and 24 (nasal side of the visual field 21 × 3 deg and 27 × 3 deg). *m* and *b* represent the slope and intercept of the linear relationship relating ganglion cell count and visual field sensitivity at a given *ec* value. *s* refers to visual acuity at a certain locus of the visual field in dB. *d* refers to the axonal density (axons/μm^2^). *c* is highly related to the severity of the disease and is used to correct the ratio of axonal to nonaxonal cell composition of RGC cells in the model.

Considering that the mild manifestation of glaucoma derived mainly from RNFL damage while the severe stage reveals a more variable MD damage in the visual field, Medeiros et al. (15–17) performed a weighted integration by which the model relates structure to function.


$$\begin{array}{l} Estimate\;RGC\;count=\left(1+MD/30\right)\times OCTrgc\\ \;+\left(-MD/30\right)\;\times SAPrgc \end{array}$$

### The Original Model for an Estimate of Macular Ganglion Cell Count

Hood et al. found that mild glaucoma damages were detected in the macula (21, 22). Based on the previous model for eRGC estimation, Medeiros et al. (18) have modified the visual function test. The scattered loci in the Humphrey 24-2 SAP were changed to focused 16 loci of the retinal sensitivity within 10° of the macular fixation areas.


$$\begin{array}{l} m=\left[0.054\left(ec\times 1.32\right)\right]+0.9\\ b=\;\left[\;-1.5\left(ec\times 1.32\right)\right]-14.8\\ gc=\left\{\left[\left(s-1\right)-b\right]/m\right\}+4.7\\ macular\;SAPrgc=\sum 10ˆ\left(gc\times 0.1\right)\\ d=\left(-0.007\times age\right)+1.4\\ c\;=\left(-0.26\times TD\right)\;+0.12\\ a\;=average\;temporal\;RNFL\;thickness\times 0.51\;\times \;10,870\times d\\ macular\;OCTrgc=10ˆ\left\{\left[\log\left(a\right)\times 10-c\right]\;\times 0.1\right\} \end{array}$$

In the above formulas, *ec* was denoted as 4.2 (3 × 3 deg), 9.5 (3 × 9 deg and 9 × 3 deg), and 12.8 (9 × 9 deg). TD is the mean value of retinal sensitivity at 16 loci corresponding to 10° of the center of total deviation using 24-2 SAP. Corresponding estimated RGC count, namely, the macular *SAPrgc* was obtained by applying the value of each point detected by SAP to the above formula. Similarly, structural parameters were changed to mean RNFL of the temporal, superior, and inferior temporal regions of the optic disc corresponding to the macula.

The aforementioned formulas gave the OCT measurements in the macula, namely, *macular OCTrgc*. The formulas were weighted and integrated according to the previous description by Medeiros et al. and the obtained RGC remained both structural and functional components.


$$\begin{array}{l} mRGC2=\left(1+MD/30\right)\times mOCTrgc+\left(-MD/30\right)\;\times mSAPrgc \end{array}$$

### A New Model for RGC Estimation in the Macula

Three innermost layers of the retina are preferentially involved when there is glaucomatous damage in the macular: the nerve fiber layer, the ganglion cell layer, and the inner plexiform layer. These three layers contain ganglion cell axons, ganglion cell body, and ganglion cell dendrites, which are collectively called the GCC. The latest frequency-domain OCT uses Fourier technology and broadband light source technology to acquire and process data, which has higher resolution, sensitivity, and faster scanning speed than time-domain OCT, and can acquire and analyze the structural images and data information of GCC in the macula in a short time. The estimation model built by Harwerth and Medeiros was adopted in the modified macular structure-function model. SAP test was changed to Humphrey 10-2 program which focuses on 16 loci within 10° of the macula (2° interval). *ec* was taken as 1.4 (1 × 1 deg), 4.2 (3 × 3 deg), 7.0 (5 × 5 deg), and 8.6 (7 × 5 deg). Macular SAPrgc, corresponding to the estimated RGC count was obtained by applying the value of each point detected by SAP to the formula.


$$\begin{array}{l} m=\left[0.054\left(ec\times 1.32\right)\right]+0.9\\ b=\;\left[\;-1.5\left(ec\times 1.32\right)\right]-14.8\\ gc=\left\{\left[\left(s-1\right)-b\right]/m\right\}+4.7\\ macular\;SAPrgc=\sum 10ˆ\left(gc\times 0.1\right) \end{array}$$

Ganglion cell complex thickness of the macula was taken as the structural parameter. MD is the value used for the center of the visual field in Humphrey SAP 10-2 program (interval 2°). Macular OCTrgc was obtained by the OCT measurements of the macula for RGC estimates.


$$\begin{array}{l} \;d=\left(-0.007\times age\right)+1.4\\ \;c\;=\left(-0.26\times TD\right)\;+0.12\\ a=average\;macular\;GCC\;thickness\times 10,870\times d\\ macular\;OCTrgc=10ˆ\left\{\left[\log\left(a\right)\times 10-c\right]\;\times 0.1\right\} \end{array}$$

The formula for modified macular SAPrgc is the same used in the above model for RGCs estimate.


$$\begin{array}{l} mRGC1=\left(1+MD/30\right)\times mOCTrgc+\left(-MD/30\right)\;\times mSAPrgc \end{array}$$

### Statistical Analysis

Continuous variables were checked to meet the normality conditions of the Shapiro-Wilk test. ANOVA test and the least significant difference *post-hoc* test with Bonferroni adjustment were used for intergroup comparisons for normally distributed variables. Dichotomic variables were analyzed using the χ^2^ test or the Fisher's exact test. Receiver operating characteristic (ROC) curve analyses were used to assess the diagnostic accuracy of eRGC, mRGC1, and mRGC2. The area under the ROC curve (AUC), sensitivity, specificity were calculated to assess the performance of the prediction model. Yorden's index, defined as the sensitivity + specificity – 1, was used to determine the optimal cut-off values to maximize diagnostic efficiency. Statistical significance was set at *P* ≤ 0.05. By setting the alpha to 0.05 to declare the slope of RGC loss as statistically significant, we were able to maintain a specificity of 95%, as demonstrated in the previous study that estimated rates of RGC loss obtained from SAP and OCT (16). At 95% specificity, approximately six of the 119 eyes would be expected to show significant slopes just by chance. The SPSS program package version 21.0 (Statistic Package for the Social Science, Inc., Chicago, IL, USA) was used for statistical analysis.

## Results

### Comparison of the Demographic and Clinical Characteristics of Healthy Eyes and POAG Eyes in Different Stages

A total of 89 eyes with POAG meeting the diagnostic criteria were enrolled. Healthy control included 30 eyes of healthy examined subjects. According to the AGIS scoring system, POAG eyes were divided using Humphrey SAP 24-2 program, namely, 20 eyes with preperimetric glaucoma, 20 eyes with the mild stage of glaucoma, 20 eyes with moderate stage, and 29 eyes with advanced stage. The demographic and clinical characteristics of subjects among the groups were shown in Table 1. The glaucomatous eyes have significantly worse visual field index (VFI), MD (24-4 and 10-2), GCC, RNFL, eRGC, mRGC2, and mRGC1 than healthy eyes (*P* < 0.001).

**Table 1:** Demographic and clinical characteristics of healthy eyes and primary open-angle glaucoma (POAG) eyes in different stages.

**Variables**	**Healthy**	**Glaucoma**				***P* value**
	**(*n* = 30)**	**PPG (*n* = 20)**	**Mild (*n* = 20)**	**Moderate (*n* = 20)**	**Severe (*n* = 29)**	
Age (years)	48.2 ± 12.6	46.4 ± 10.2	48.1 ± 15.2	50.5 ± 18.5	48.9 ± 14.1	0.921
VFI(%)	98.2 ± 1.9	97.2 ± 2.4	90.2 ± 4.9	65.6 ± 9.3	24.0 ± 17.4	0.000
MD24-2(dB)	−1.54 ± 0.97	−2.59 ± 1.26	−4.82 ± 2.11	−14.85 ± 3.44	− 26.73 ± 4.41	0.000
MD10-2(dB)	−0.37 ± 0.81	−0.63 ± 1.40	− 3.78 ± 2.48	−9.57 ± 4.30	− 21.37 ± 8.29	0.000
GCC(um)	105.95 ± 4.83	92.38 ± 4.10	84.04 ± 8.98	71.23 ± 7.63	61.40 ± 6.37	0.000
RNFL(um)	110.37 ± 4.90	95.65 ± 6.34	87 ± 9.40	72.60 ± 10.94	58.76 ± 11.93	0.000
mRGC1	11,739,81 ± 77,580	10,189,84 ± 60156	8,487,95 ± 110,610	6,428,02 ± 157,538	2,272,90 ± 42,206	0.000
mRGC2	6,195,39 ± 58,775	5,293,83 ± 37,422	4,087,60 ± 67,796	2,322,21 ± 56,410	921,18 ± 80978	0.000
eRGC	11,021,08 ± 72,669	8,939,76 ± 87,103	6,809,84 ± 118,903	2,897,88 ± 96,051	822,68 ± 72,653	0.000

*eRGC, estimated retinal ganglion cell; mRGC, macular estimated retinal ganglion cell; GCC, ganglion cell complex; MD, mean deviation; POAG, primary open-angle glaucoma; PPG, preperimetric glaucoma; RNFL, retinal nerve fiber layer; VFI, visual field index*.

### Comparison of the Three Estimated Values for Each Group

**Figure 1:**
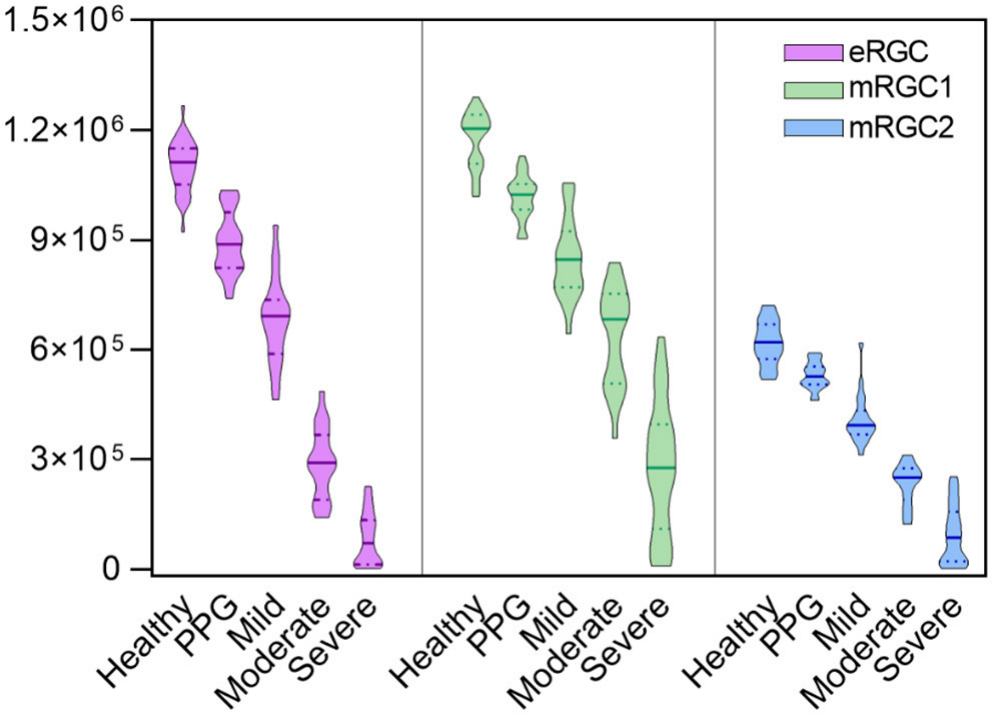
Violin plots illustrating that estimated retinal ganglion cell (eRGC), macular estimated retinal ganglion cell (mRGC1), and mRGC2 were obtained from the three models at five stages. It showed that eRGC, mRGC1, and mRGC2 decreased markedly from preperimetric glaucoma (PPG) to severe stage of glaucoma (*P* < 0.05).

Compared with normal individuals (Figure 1), eRGC, mRGC1, and mRGC2 obtained from the three models decreased markedly in glaucoma, and as glaucoma progressed, RGC estimates reduced gradually from mild to severe stage.

Compared with the estimated RGCs in healthy eyes, preperimetric glaucomatous eyes had mRGC1 reduced to 13.2%, mRGC2 reduced to 14.5%, and eRGC reduced to 18%. Glaucomatous eyes with mild stages had mRGC1 reduced to 28%, mRGC2 reduced to 34%, and eRGC reduced to 38%. In the severe stage, the reduction proportion for mRGC1, mRGC2, and eRGC was 81, 85, and 92%, respectively, indicating that mRGC1 has a good staging performance and may serve as a candidate model for estimating RGC counts in the macula.

### Diagnostic Efficacy and Sensitivity Analysis of eRGC, mRGC1, and mRGC2 and Related Structural/Functional Parameters

**Figure 2:**
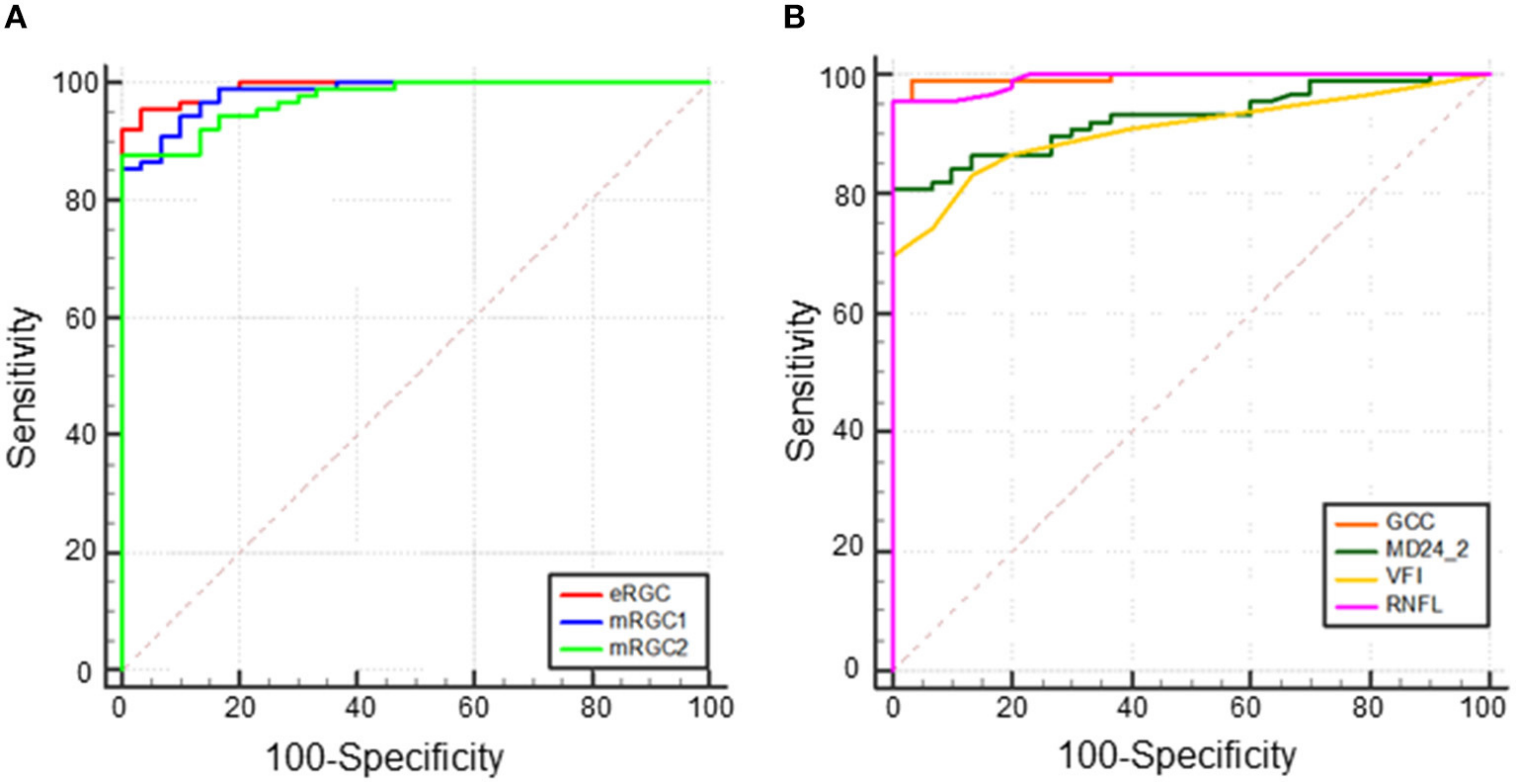
Receiver operating characteristic (ROC) curve of total eRGC, mRGC1, and mRGC2 and structural/functional parameters in POAG, **(A)** Area under the ROC curve (AUC) of eRGC, mRGC1, and mRGC2 was 0.992 (95% CI, 0.954~1.000), 0.982 (95% CI, 0.939~0.998), 0.972 (95% CI, 0.924~0.994), respectively, and **(B)** AUC of ganglion cell complex (GCC), retinal nerve fiber layer (RNFL), visual field index (VFI), and MD (24-2) was 0.995 (95% CI, 0.960~1.000), 0.992 (95% CI, 0.954~1.000), 0.906 (95% CI, 0.839–0.952), and 0.927 (95% CI, 0.865–0.967), respectively.

To analyze the capability of eRGC, mRGC1, and mRGC2 and also structural parameters (GCC and RNFL) and functional parameters (VFI and MD24-2) in differentiating between POAG and healthy eyes, ROC curves were conducted. The results showed that the AUCs of all parameters were above 0.9. As shown in Figure 2, AUC of eRGC, mRGC1, and mRGC2 was 0.992 (95% CI, 0.954~1), 0.982 (95% CI, 0.939~0.998), and 0.972 (95% CI, 0.924~0.994), respectively. The AUC of structural parameter GCC was 0.995 (95% CI, 0.960~1) and the AUC of peripapillary RNFL was 0.992 (95% CI, 0.954~1). The AUCs for the functional parameters VFI and MD (24-2) were 0.906 (95% CI, 0.839–0.952) and 0.927 (95% CI, 0.865–0.967), respectively. All these indicators offered relatively good predictive performances to distinguish glaucoma from a healthy population.

For a 95% specificity as shown in Table 2, eRGC had a sensitivity of 95.51%, with a relatively low sensitivity of 86.52 and 87.64% for mRGC1 and mRGC2, respectively. The sensitivity of structural parameters GCC and RNFL were 98.88 and 95.51%, respectively, while the sensitivity of the functional parameter MD24-2 is 80.90%, the VFI sensitivity is 73.03%, much lower than the sensitivity of mRGC1 and mRGC2.

**Table 2:** Diagnostic efficacy and sensitivity analysis of eRGC, mRGC1, and mRGC2 and related structural/functional parameters.

**Variables**	**Sensitivity at 95% specificity**	**AUC (95% CI)**	**Yorden index**	***P* value**
eRGC	95.51%(87.64~100.00%)	0.992(0.954~1.000)	0.9217	<0.0001
mRGC1	86.52%(74.16~94.38%)	0.982(0.939~0.998)	0.8539	<0.0001
mRGC2	87.64%(78.65~93.26%)	0.972(0.924~0.994)	0.8764	<0.0001
GCC(um)	98.88%(91.01~100.00%)	0.995(0.960~1.000)	0.9554	<0.0001
RNFL(um)	95.51%(88.76~98.88%)	0.992(0.954~1.000)	0.9551	<0.0001
MD24-2(dB)	80.90%(69.66~87.64%)	0.927(0.865~0.967)	0.8090	<0.0001
VFI(%)	73.03%(56.68~83.15%)	0.906(0.839~0.952)	0.6981	<0.0001

*eRGC, estimated retinal ganglion cell; GCC, ganglion cell complex; mRGC, macular estimated retinal ganglion cell; RNFL, retinal nerve fiber layer; MD, mean deviation; VFI, visual field index*.

## Discussion

Previous studies have built a model that combines structure and function measures to estimate RGC losses and validated it in both experimental studies and human glaucoma (5, 14, 19). Along with this, the involvement of the macula has been evidenced in mild glaucomatous damage (21). However, the current assessment of structural damage in glaucoma is mainly based on damages to the optic disc and peripapillary RNFL, few studies have investigated the clinical significance of macular damage for diagnosis and staging in glaucoma. The original model for macular RGC estimation structurally applied the temporal RNFL thickness of the optic disc and functionally applied 16 central points of the 24-2 SAP test corresponding to the central 10° to assess macular RGC loss. However, there are two limitations in this model: the first is that the temporal RNFL of the optic disc does not reflect the real situation of ganglion cell damage in the macula. Second, approximately 50% of RGCs are located within 16° of the central macula, an area that accounts for only 7.3% of the total retinal area. In the SAP test 24-2 program, only 12 (22%) of the 54 loci (6° interval) were located in this region, so mild glaucomatous visual field defects may be missed and also a large difference in the real number of residual RGCs in the macula was observed. Therefore, in this study, we made adjustments to the original model and sought to propose a novel improved macular RGC estimation model with the combination of both structural and functional measurements. The structural parameter for temporal peripapillary RNFL thickness measurement was changed to the macular structural parameter GCC thickness. The SAP testing program and location were changed from 24-2 with a large range (interval 6°) to 10-2, focusing on visual sensitivity that has 16 loci within 10° of the macular fixation points (interval 2°).

Table 1 and Figure 1 showed that eRGC, mRGC1, and mRGC2 were significantly higher in normal subjects than in patients with glaucoma and that the eRGC decreased gradually in patients with glaucoma as the disease progressed, with statistically significant differences between all groups. eRGC, mRGC1, and mRGC2 provided good performance in discriminating glaucomatous from healthy eyes, and different degrees of severity or progression. Compared with the normal controls, mRGC1, mRGC2, and eRGC were reduced by 13.2, 14.5, and 18%, respectively, in preperimetric glaucoma. In mild glaucoma, mRGC1, mRGC2, and eRGC were reduced by 28, 34, and 38%, respectively. In the advanced disease, mRGC1, mRGC2, and eRGC were reduced by 81, 85, and 92%, respectively. The proportion of RGC loss in the macula was close to the proportion outside the macula in different stages of POAG, indicating that the macular ganglion cell damage started at early stages, and as the disease progresses, parallel damages would be observed both in the macula and outside the macula. In the preperimetric stage, there was already about 13.2% (14.5% for mRGC2 estimation) RGC loss in the macula and the decline of MD in SAP 10-2 was 0.63 dB, 0.3 dB lower than normal controls; however, in the progression stage, there was a considerate loss of macular and eRGC estimations, and the MD declined significantly, suggesting that SAP was not sensitive in the detection of mild glaucoma. While the macular had the highest RGC density, thus in the early stage of disease, a significant reduction of mRGC1 and mRGC2 could be observed, which is helpful in the early diagnosis.

Several studies have revealed that RGC estimates that integrate structural and functional parameters have higher diagnostic efficacy for glaucoma than structural or functional parameters applied alone. Medeiros et al. (23) found that RGC estimates were superior to mean RNFL thickness in distinguishing glaucomatous from healthy eyes (AUCs 0.95 and 0.88, respectively). Our data demonstrated that the AUCs of mRGC1, mRGC2, and eRGC in the three RGC estimation models of POAG were 0.982 (95% CI, 0.939–0.998), 0.972 (95% CI, 0.924–0.994), and 0.992 (95% CI, 0.954–1.000), respectively, which were higher than those of the separate functional AUC 0.906 (95% CI, 0.839~0.952) and 0.927 (95% CI, 0.865~0.967) for VFI and MD (24-2). At 95% specificity, eRGC had a sensitivity of 95.51%, and the sensitivity of mRGC1 and mRGC2 estimates were relatively low, with 86.52 and 87.64% respectively. The macular region analyzed in the macular structure-function model may contain only about 50% of RGCs, while the RNFL in the conventional optic disc-visual field model contains axons of ganglion cells in a wide area of the retina and can detect glaucomatous damage in more locations outside the macula, resulting in higher diagnostic efficacy for eRGC. For a 95% specificity, the structural parameter GCC was 98.88%, significantly higher than the functional parameters MD (24-2) with a sensitivity of 80.90% and VFI with a sensitivity of 73.03%. In addition, because the macular region is less affected by blood vessels during OCT imaging, thus making GCC is an ideal tool for glaucoma detection.

It is also worth noting that there are some limitations to this study. The number of RGCs is based on models and formulas from SAP and OCT data, not directly derived from histologic data. Although the formula has been validated in animals and glaucoma populations, there are still some deviations from the real RGCs number. Furthermore, differences in detection equipment and individual variability in disc-macula structures should also be taken into account, which may indirectly or directly affect the accuracy of macular RGCs count estimation. Another limitation is that the macular estimated RGC count could be affected by macular diseases. The macular examination is, therefore, required to exclude any macular affection. Future prospective studies are needed to further validate our macular structure-function model for estimating the number of macular RGCs.

In conclusion, this study performed a comparative analysis for three RGC estimation models. Our results showed that mRGC1, mRGC2, and eRGC all provided good performances in the severity grading of glaucoma. No significant differences were observed in diagnostic efficacy between the new modified macular structure-function models mRGC1 and eRGC. Meanwhile, the results of GCC were more stable with fewer individual differences. Thus, our model combining structure and function is expected to be a new strategy for the evaluation of glaucomatous damage in the macula and has significant clinical implications regarding the diagnosis and early detection of glaucoma progression.

## Data Availability Statement

The raw data supporting the conclusions of this article will be made available by the authors, without undue reservation.

## Ethics Statement

The studies involving human participants were reviewed and approved by the Ethics Committee of The First Affiliated Hospital of Kunming Medical University. The patients/participants provided their written informed consent to participate in this study.

## Author Contributions

YW, HZ, and QC designed the study and wrote the manuscript. YW, QC, YT, WY, JW, and DF collected data of the patients and performed various clinical examinations. YW, YZ, and QC analyzed the data. HZ gained the fund and supervised the process. All the authors approved the final version of the article.

## Funding

This study was supported by the National Natural Science Foundation of China under grant No. 81760170, Yunnan health training project of high level talents L-2017004, Science and Technology Innovation Team of Ophthalmology of Kunming Medical University CXTD201902, Yunnan Province “High-level Talents Training Support Plan” Famous Doctor Special Project.

## Conflict of Interest

The authors declare that the research was conducted in the absence of any commercial or financial relationships that could be construed as a potential conflict of interest.

## Publisher's Note

All claims expressed in this article are solely those of the authors and do not necessarily represent those of their affiliated organizations, or those of the publisher, the editors and the reviewers. Any product that may be evaluated in this article, or claim that may be made by its manufacturer, is not guaranteed or endorsed by the publisher.
